# Supplementary figures and images for: Single cell analyses reveal distinct adaptation of typhoidal and non-typhoidal Salmonella enterica serovars to intracellular lifestyle
Source: PLoS Pathog. 2021 Jun 18;17(6):e1009319. doi: 10.1371/journal.ppat.1009319 (PMC8244875; doi:10.1371/journal.ppat.1009319)

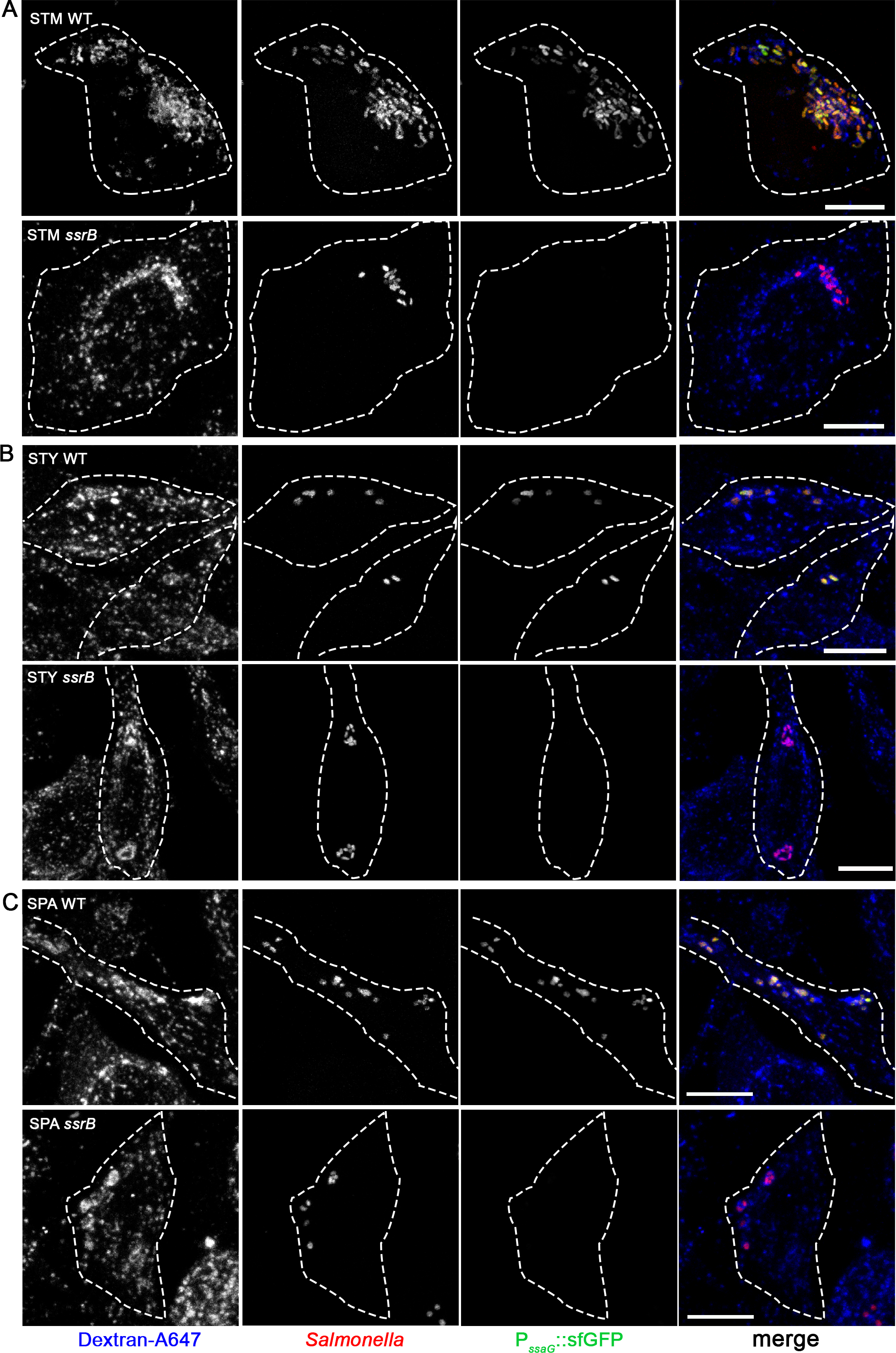

Supplement: S1 Fig — HeLa cells were infected at MOI 75 with STM (A), STY (B), or SPA (C), WT or ΔssrB strains all harboring plasmid p3776 for constitutive expression of RFP (red), and sfGFP (green) under control of the ssaG promoter. To label the endosomal system, cells were pulsed with Dextran-Alexa647 (Dextran-A647, blue) from 1 h p.i. until fixation. Cells were fixed with PFA 8 h p.i. and microscopy was performed by CLSM on a Leica SP5 using the 100x objective. Scale bars, 10 μm. (TIF) [file ppat.1009319.s001.tif]

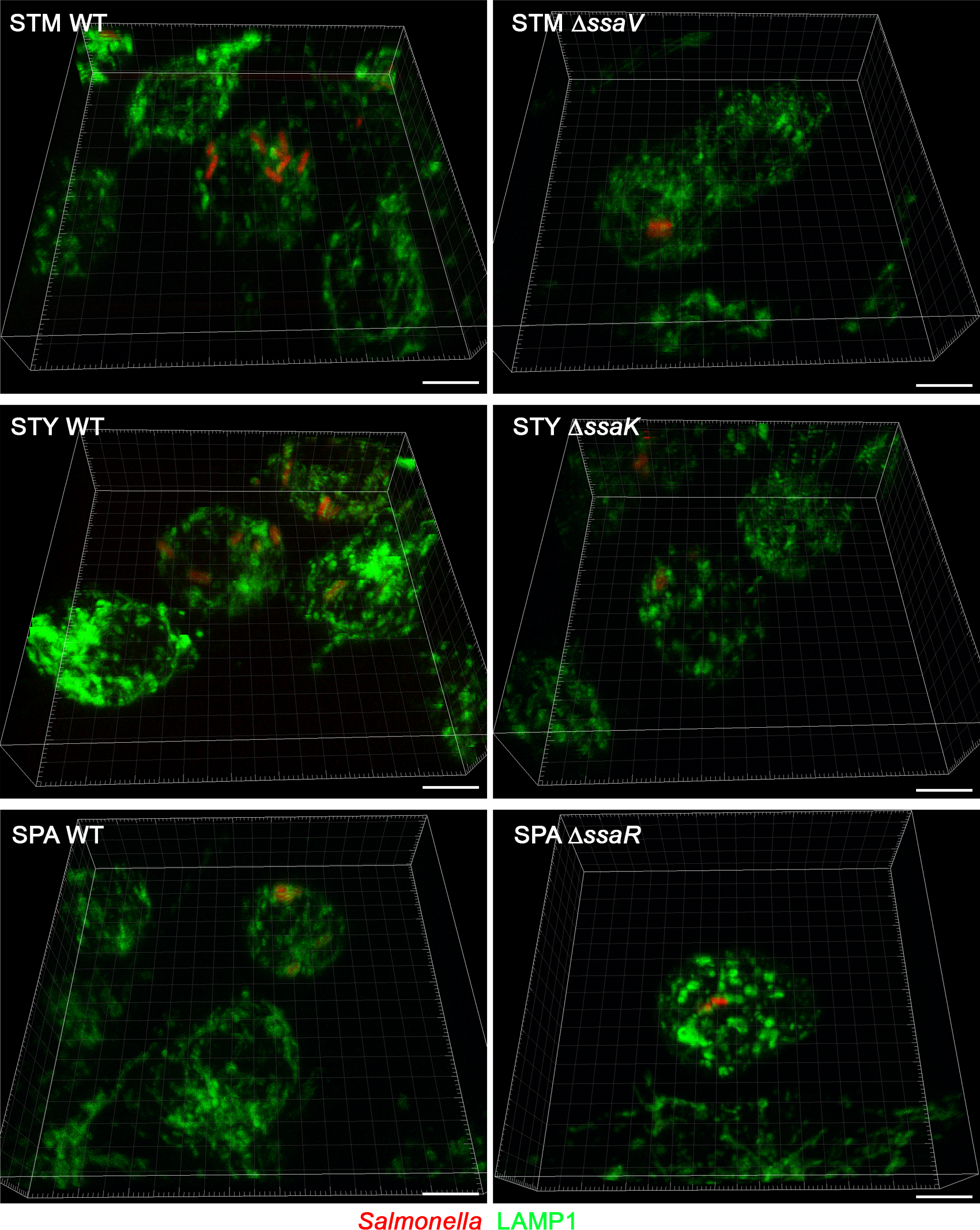

Supplement: S2 Fig — RAW264.7 cells constitutively expressing LAMP1-GFP, without IFN-γ-activation, were infected by WT or SPI2-T3SS-deficient strains (ΔssaV, ΔssaK, or ΔssaV) of STM, STY, or SPA as indicated, each constitutively expressing mCherry. Cells were fixed 8 h p.i. and subjected to CLSM on a Leica SP5. 3D reconstructions of Z stacks are shown. Scale bars, 5 μm. (TIF) [file ppat.1009319.s002.tif]

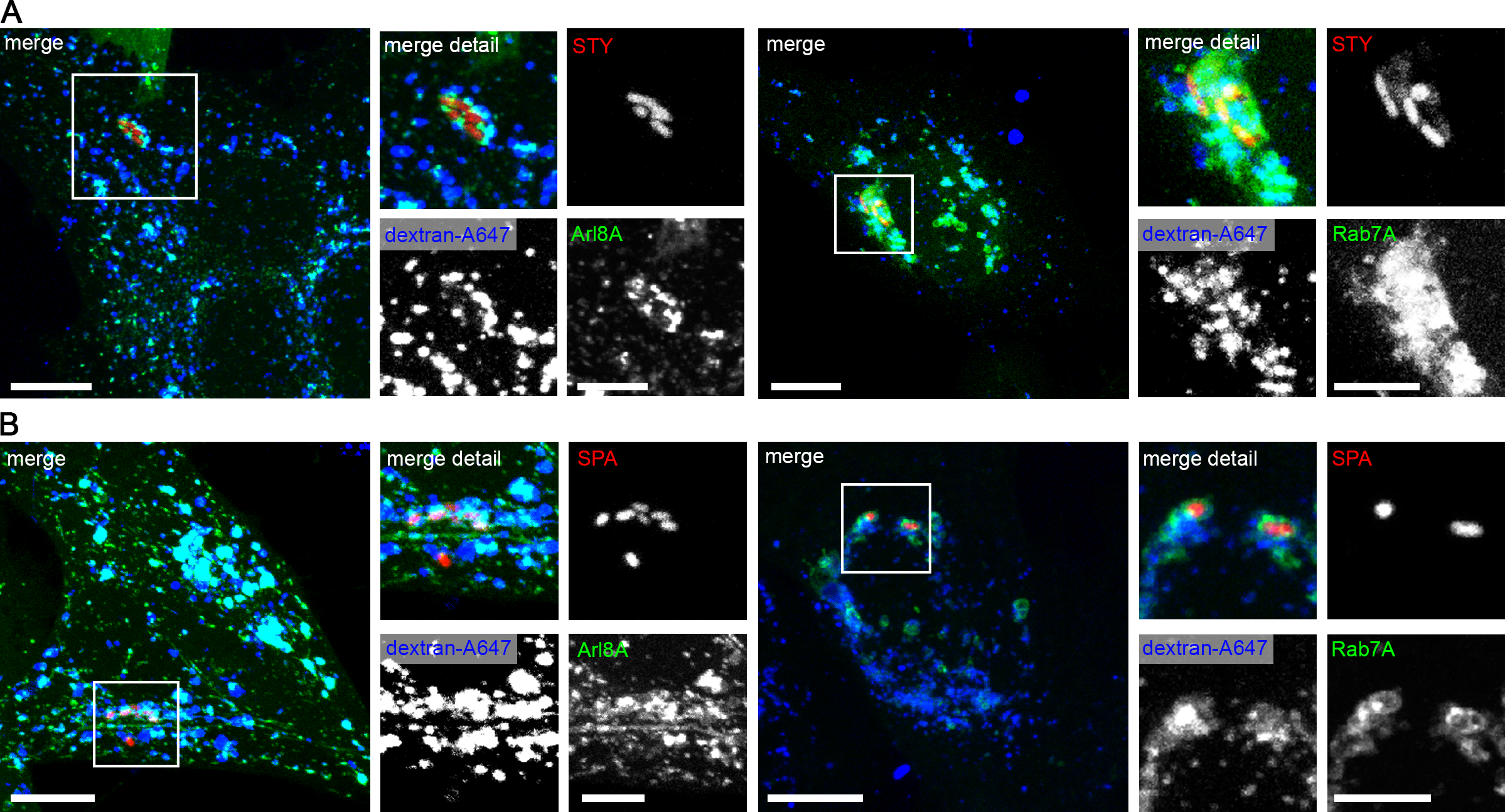

Supplement: S3 Fig — HeLa cells were transiently transfected with plasmids for the expression of Arl8A-eGFP or Rab7A-eGFP as indicated, and infected with STY (A) or SPA (B) at MOI 75 (for STY) or 50 (for SPA). Infected cells were pulsed-chased with Dextran-Alexa647 from 1 h p.i. until fixation. Microscopy was performed by CLSM on a Leica SP5 using a 100 x objective. Scale bars, 10 μm (overview); 5 μm (detail). (TIF) [file ppat.1009319.s003.tif]

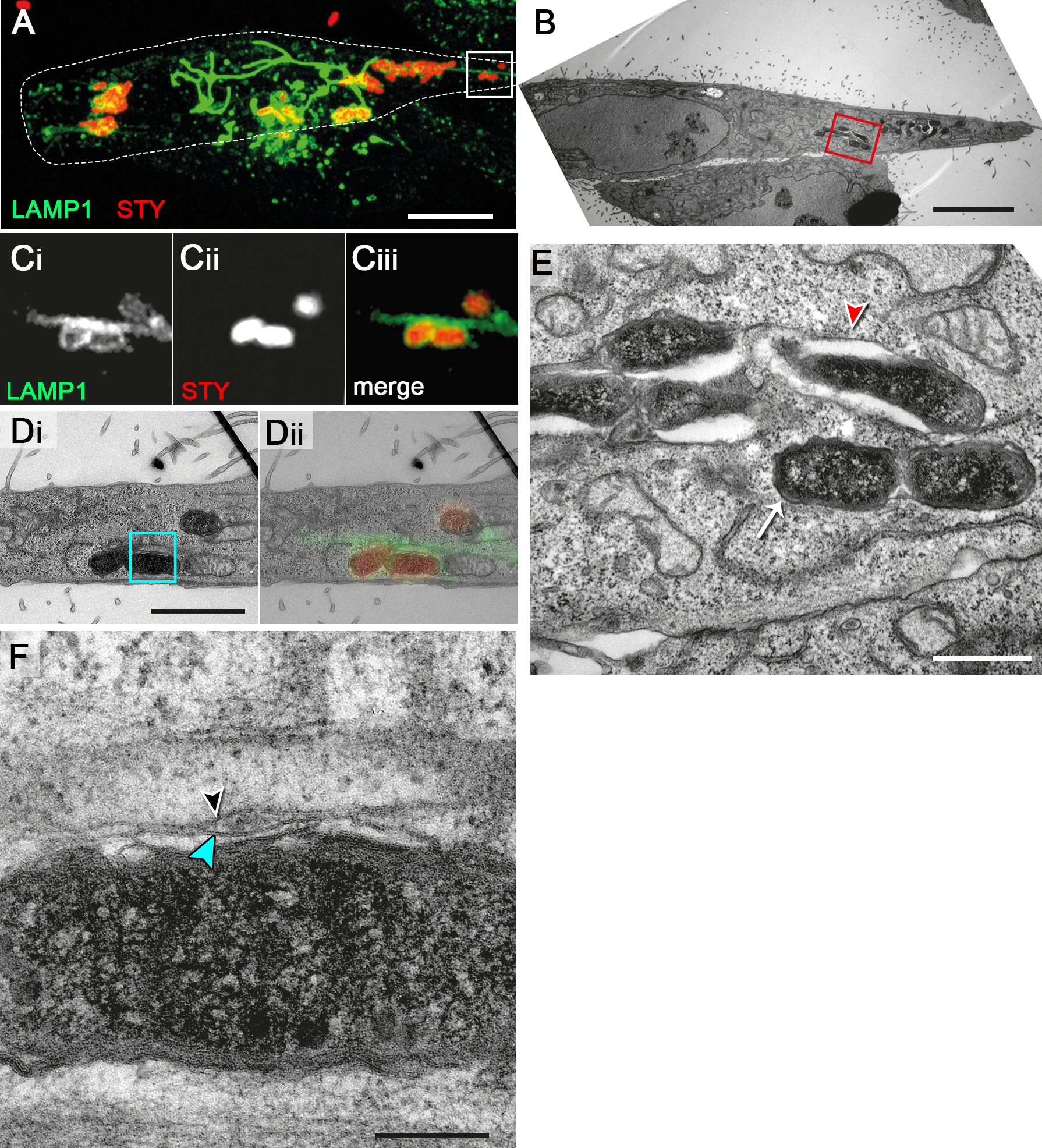

Supplement: S4 Fig — HeLa cells expressing LAMP1-GFP (green) were infected with STY WT expressing mCherry (red). Cells were fixed 7 h p.i. and processed for confocal microscopy (A, C) and TEM of ultrathin sections (B, D, E, F). A) An infected cell showing a distinct LAMP1-GFP-positive SIF network was identified, further analyzed, ad is shown in maximum intensity protection (MIP). B) TEM overview image of a host cell harboring several salmonellae. Ci, ii, iii, Di, ii) For correlation, TEM and CLSM images were superimposed. E) Detail of individual STY cells in SCV with single membrane (red arrowhead, white arrow). F) Detail view of a small section of a double membrane SIF with inner (black arrowhead) and outer (blue arrowhead) membrane. Scale bars: 10 μm (A), 7 μm (B), 2 μm (D), 750 nm (E), 200 nm (F). (TIF) [file ppat.1009319.s004.tif]

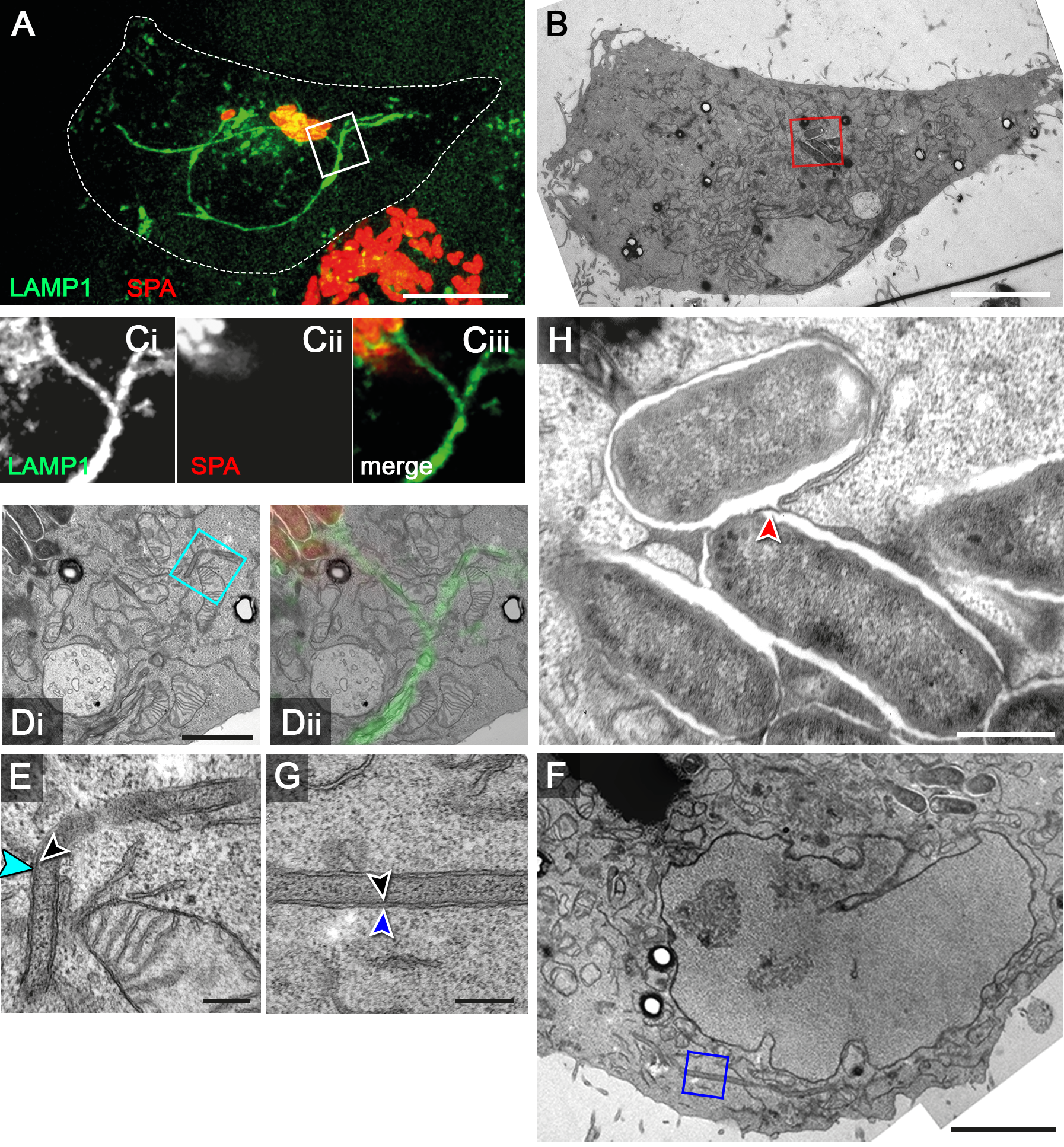

Supplement: S5 Fig — HeLa cells expressing LAMP1-GFP (green) were infected with SPA WT expressing mCherry (red). Cells were fixed 7 h p.i. and processed for confocal microscopy (A, C) and TEM of ultrathin sections (B, D, E, F, G, H). A) An infected cell showing a distinct LAMP1-GFP-positive SIF network was identified, further analyzed and shown as MIP. B) TEM overview image of a host cell harboring several salmonellae (red box). Ci, ii, iii, Di, ii) For correlation, TEM and CLSM images were superimposed. H) Detail of individual SPA cells in SCV with single membrane (red arrowhead). D, F) Overviews showing SIF distal to the SCV, regions shown in details are indicated by light or dark blue boxes. E, G) Corresponding detail views of double membrane SIF with inner (black arrowhead) and outer (light blue, dark blue arrowheads) membranes. Scale bars: 10 μm (A), 7 μm (B), 2 μm (D), 250 nm (E, G), 3 μm (F), 500 nm (H). (TIF) [file ppat.1009319.s005.tif]

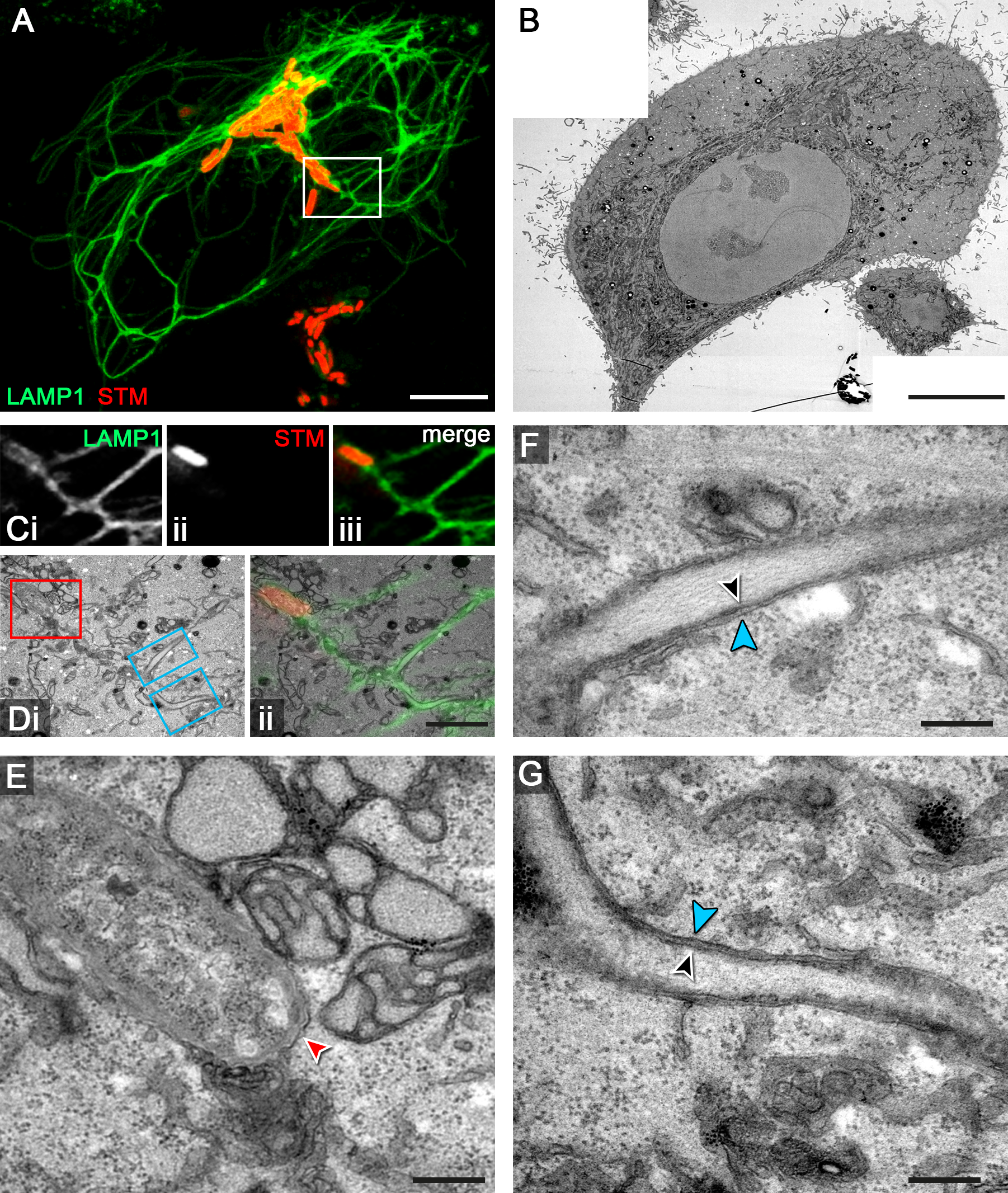

Supplement: S6 Fig — HeLa cells expressing LAMP1-GFP (green) were infected with STM WT expressing mCherry (red). Cells were fixed 7 h p.i. and processed for confocal microscopy (A, C) and TEM of ultrathin sections (B, D, E, F, G). A) An infected cell showing a distinct LAMP1-GFP-positive SIF network was identified, further analyzed and shown as MIP. B) TEM overview image of a host cell harboring several salmonellae. Ci, ii, iii, Di, ii) For correlation, TEM and CLSM images were superimposed. E) Detail of individual STM cells in SCV with single membrane (red arrowhead, white arrow). F, G) Detail views of small sections of double membrane SIF with inner (black arrowhead) and outer (blue arrowhead) membrane. Scale bars: 10 μm (A), 7 μm (B), 2 μm (D), 750 nm (E), 200 nm (F, G). (TIF) [file ppat.1009319.s006.tif]

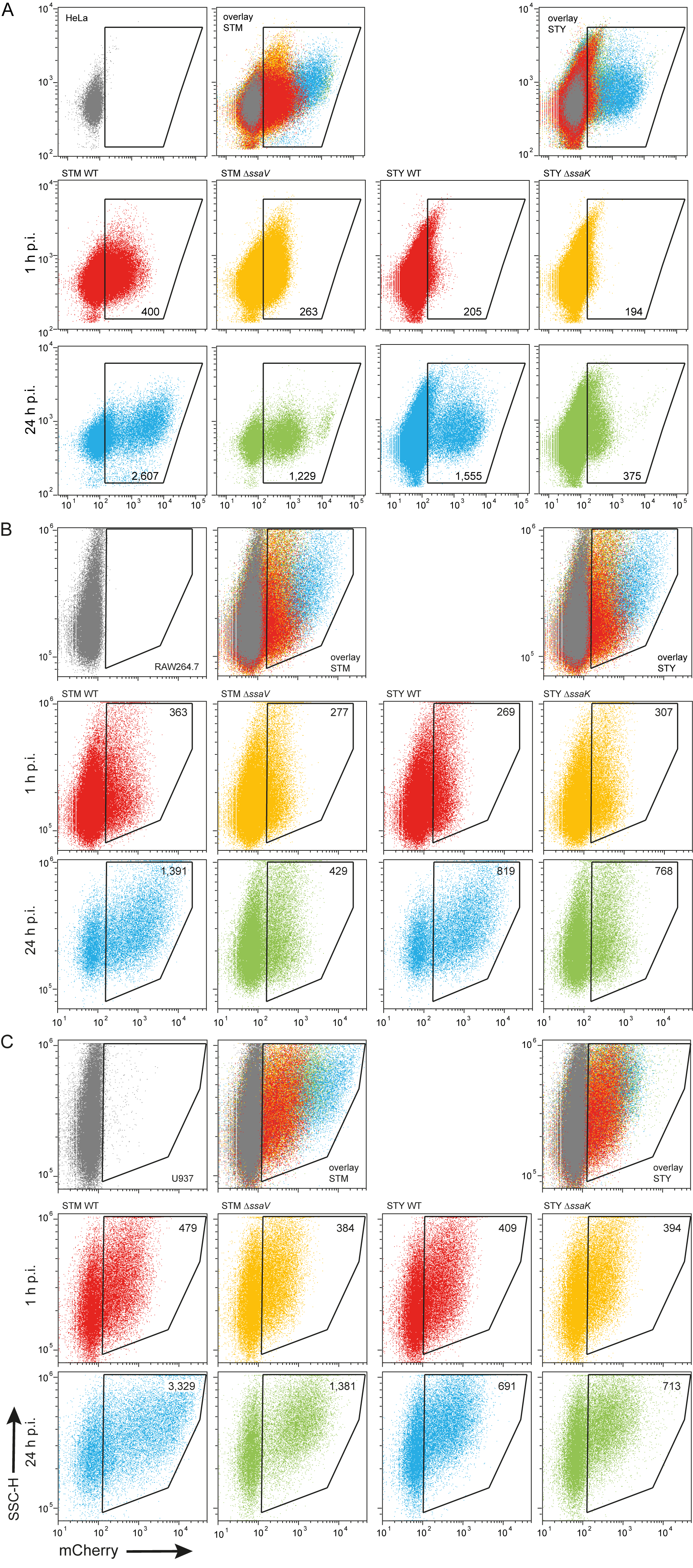

Supplement: S7 Fig — The single dot plots of the experiment shown in Fig 8 are displayed. HeLa cells (A), RAW264.7 macrophages without IFN-γ-activation (B), or U937 macrophages without IFN-γ-activation (C) were used as host cells. Gating for infected host cells was made using non-infected cells (grey dots) as control. Overlays of dot plots show WT Salmonella at 1 h or 24 h p.i. in red and blue, respectively, and ΔssaV or ΔssaK Salmonella at 1 h or 24 h p.i. in orange and green, respectively. The X-mean values for mCherry fluorescence intensities of infected cells are indicated the gates for infected host cells. Assays were performed in three biological replicates, one representative replicate is shown. (TIF) [file ppat.1009319.s007.tif]

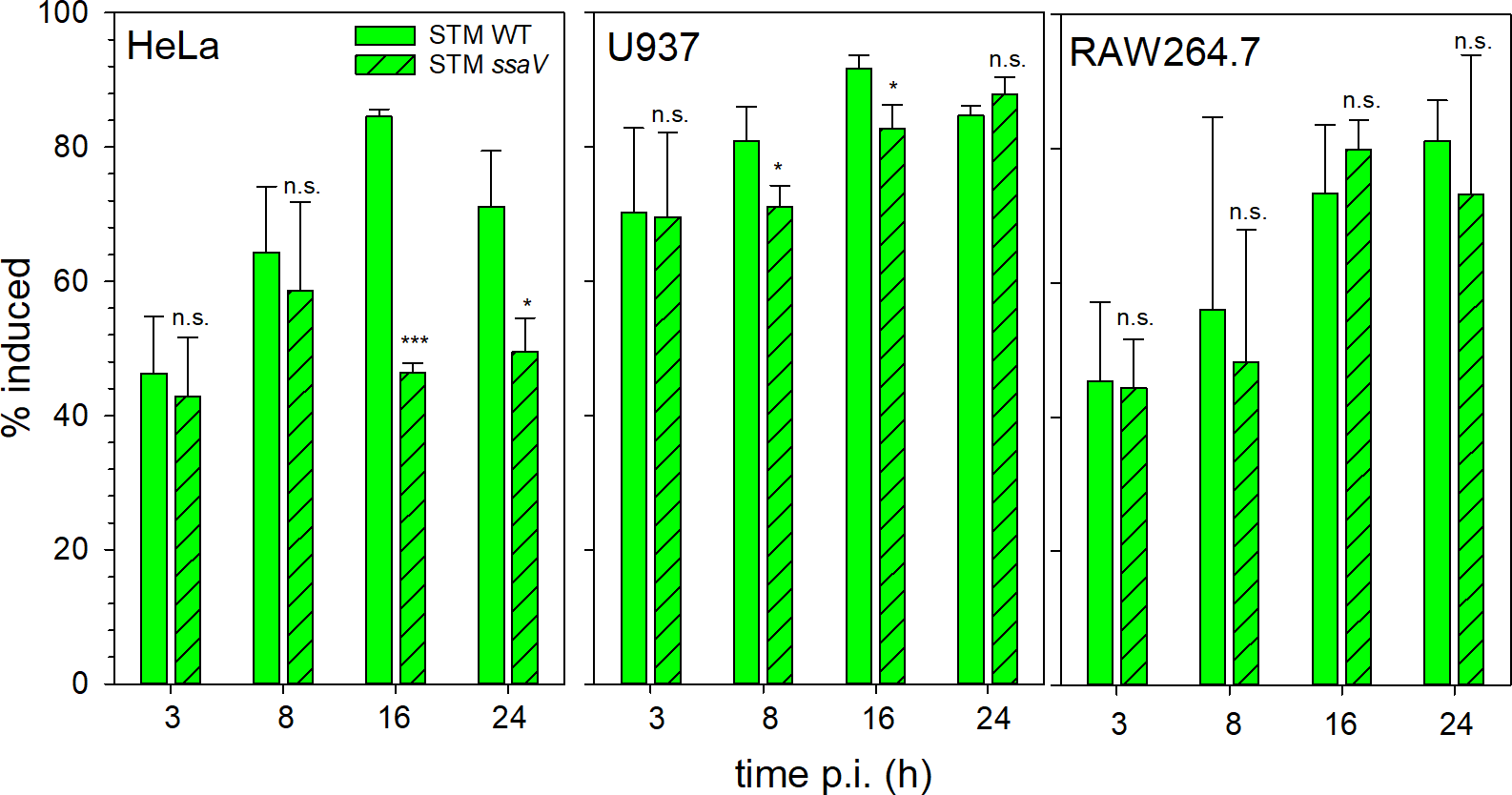

Supplement: S8 Fig — STM (green) WT (open bars) and SPI2-T3SS-deficient ΔssaV (hatched bars) strains, each harboring plasmid p4928 were used to infect HeLa cells, U937 macrophages without IFN-γ-activation, or RAW264.7 macrophages without IFN-γ-activation as indicated. Infection, induction of expression and analyses by FC were basically performed as described for Fig 9. Host cells were lysed at the indicated time points, bacteria-sized particles were gated based on DsRed fluorescence, and the sfGFP-positive population was gated. The means and standard deviations of sfGFP-induced Salmonella of three biological replicates are shown. Statistical analyses were performed as described for Fig 9. (TIF) [file ppat.1009319.s008.TIF]
