# Supplementary material for: Single cell analyses reveal distinct adaptation of typhoidal and non-typhoidal Salmonella enterica serovars to intracellular lifestyle
Source: PLoS Pathog. 2021 Jun 18;17(6):e1009319. doi: 10.1371/journal.ppat.1009319 (PMC8244875; doi:10.1371/journal.ppat.1009319)
Supplement: S1 Table — (DOCX) [file ppat.1009319.s009.docx]

**Oligonucleotides used in this study**

#### Designation Sequence 5’ – 3’

#### Red mutagenesis primer

#### ssaK-Del13-For TCTTCATGGAGCATTTGCTATGAGTTTTACTTCACTTCC TATTCCGGGGATCCGTCGACC

#### ssaK-Del13-Rev CATTACGTAACCATTTCAGTAACGCGTTGAAATGACGAG ATGTAGGCTGGAGCTGCTTCG

#### ssaR Del13 For ATAATAGCGTTCCAGGTCGTGTCATGAGAGATAC AGTATGATTCCGGGGATCCGTCGACC

#### ssaR Del13 Rev GAATCATTCATGAAAAGCTCTGTACCAATTGCGCC AGTGTTGTAGGCTGGAGCTGCTTCG

#### STY ssrB Del13 For ATATTATCTTAATTTTCGCGAGGGCAGCAAAATGAAAGAA ATTCCGGGGATCCGTCGACC

#### STY ssrB Del13 Rev ACCAATGCTTAATACCATCGGATGCCCCTGGTTAATAATAT GTAGGCTGGAGCTGCTTCG

#### Gibson assembly primer

#### Vf-p4889 ATGCGCAAAGGCGAAGAACTGTTTACCGGTGT

#### Vr-p4889 GGCCGGCATCACCGGCGCCACAG

#### 1f p4889-PsifA TGTGGCGCCGGTGATGCCGGCCGCATCCAGGCATGAAGTT

#### 1r PsifA-sfGFP TTCTTCGCCTTTGCGCATATTAATCTCACTTATACTGGAGTAA

#### Check PCR primer

#### ssaK-DelCheck-For CGTATACTTTGGCCGAAGAC

#### ssaK-DelCheck-Rev TCCTGTAACTCCTGGAGAGC

#### ssaR DelCheck For TGGTGCGTATTACACGTTGG

#### ssaR DelCheck Rev TGAGTCAAGGCCTGAACAAG

#### STY ssaR DelCheck For CCCACAGGCAATCAACTCAC

#### STY-ssaR-DelCheck-Rev2 GTGAGTTGATTGCCTGTGGG

#### STY ssrB DelCheck For GGGCAGACTGAATTGGTATG

#### STY ssrB DelCheck Rev TAGCGTGGCGGCATTGATAC
