# Supplementary material for: Single cell analyses reveal distinct adaptation of typhoidal and non-typhoidal Salmonella enterica serovars to intracellular lifestyle
Source: PLoS Pathog. 2021 Jun 18;17(6):e1009319. doi: 10.1371/journal.ppat.1009319 (PMC8244875; doi:10.1371/journal.ppat.1009319)
Supplement: S2 Table — (DOCX) [file ppat.1009319.s010.docx]

**Antisera and antibodies used in this study**

#### Antibody or antiserum Characteristics Source or reference

#### Group B Factors 1, 4, 5, 12 Rabbit anti-STM O antiserum BD Difco

#### Group D1 Factors 1,9,12 Rabbit anti-STY O antiserum BD Difco

#### Group A Factors 1,2,12 Rabbit anti-SPA O antiserum BD Difco

#### Anti-M45 Mouse anti-M45 epitope tag [1]

#### Anti-HA Rat anti-HA epitope tag Roche

#### Anti-mouse IgG Alexa 568 Goat anti-mouse IgG Alexa 568 ThermoFisher

#### Anti-rat IgG Alexa 568 Goat anti-rat IgG Alexa 568 ThermoFisher

#### Anti-rabbit IgG Cy5 Goat anti-rabbit IgG Cy5 Jackson ImmunoResearch

#### Anti-rat IgG Cy5 Goat anti-rat IgG Cy5 Jackson ImmunoResearch

**References**

1. Obert S, O'Connor RJ, Schmid S, Hearing P. The adenovirus E4-6/7 protein transactivates the E2 promoter by inducing dimerization of a heteromeric E2F complex. Mol Cell Biol. 1994;14(2):1333-46.
