# Supplementary material for: Single cell analyses reveal distinct adaptation of typhoidal and non-typhoidal Salmonella enterica serovars to intracellular lifestyle
Source: PLoS Pathog. 2021 Jun 18;17(6):e1009319. doi: 10.1371/journal.ppat.1009319 (PMC8244875; doi:10.1371/journal.ppat.1009319)
Supplement: Attachment — Submitted filename: Response to reviewers.docx [file ppat.1009319.s011.docx]

Authors’ response: We thank the reviewers for the detailed analyses of our manuscript and the thoughtful comments. These helped us very much to eliminate errors and to improve the quality of the manuscript. We also identified many points for follow-up work and we a grateful that you shared your thought on this topic.

Please find below a point-by-point response with our response in blue font.

**Part II – Major Issues: Key Experiments Required for Acceptance**

Reviewer #1:

The role of SPI-2 for typhoidal Salmonella has indeed been poorly understood. The presented data are sometimes a bit confusing and not easy to follow, even for someone relatively familiar with these infection systems, and data visualization could be improved. What makes it hard to draw general conclusions is that often the serovars behave inconsistently between the different cell models (even between U937 and RAW264.7, which are both macrophage-like cell types). The study reports observations, but falls relatively short on providing any new mechanistic/functional insights. E.g. given their findings that SPI-2 expression is heterogeneous and the proportion of SPI-2+ vs. SPI-2- intracellular bacteria very different for the typhoidal compared with the nontyphoidal serovars, a natural question to ask is what the molecular source of this heterogeneity (or its consequence) could be. The observation that SPI-2-deficient S. Typhi show a reduced intracellular proliferation rate is interesting; yet the relevance for the in vivo setting and for the outcome of disease remain unclear. In my opinion, a mechanistic/functional aspect of this kind would be required to justify publication in this journal.

Response: Our study provides a first systematic analyses of intracellular activities of typhoidal Salmonella (TS) in comparison to S. Typhimurium as standard model organisms of non-typhoidal Salmonella (NTS). The study identifies the heterogeneity of activation of SPI2-T3SS functions and the lower level of activation in TS. These novel findings will set the basis or subsequent analyses of molecular mechanisms. We consider unravelling the molecular mechanisms of utmost interest, but clearly beyond this study. Experimental work is this direction is currently in progress, for example to investigate the differences between NTS and TS in entering persister status.

Reviewer #2:

Several of the assays report the distribution of responses of STM, SPA and STY in various cell types in the form of a single representative experiment, of three replicates. Increases or decreases of each Salmonella strain is reported but how representative and the amount of variation between biological replicates is not apparent from the manuscript. Although the conclusions based on the representative data seems very clear and appropriate it is difficult to interpret the reproducibility based on the presentation of a single representative observation of the distribution of a response. This applies to the presentation of data in Figure 1, Figure 6 and Figure 9. This could be addressed by enumerating the response by gating and applying statistical analysis to the biological replicates.

Response: We added the calculations of means and standard deviations of at least three independent biological replicates for the experiments shown in Fig. 1, Fig. 6, and Fig. 9. It should be mentioned that means and SD of induced cells only insufficiently display the population heterogeneity of intracellular Salmonella, and for this reason the detailed distribution of induction level and resulting calculation in individual experiments is displayed.

Reviewer #3:

1. The authors tried to quantify the level of T3SS-2 expression (Fig. 1), SCV integrity (Fig. 6), intracellular proliferation (Fig. 8), biosynthetic capability (Fig. 9) of intracellular S. Typhimurium, S. Typhi and S. Paratyphi A by using flowcytometric analyses in order to compare among them. However, the authors don’t repeat any assays. Authors should repeat each assay and make graphs with statistics to strengthen the data.

Response: All data displayed are based on at least three independent biological replicates. We opted to show one representative data set to provide details of the population heterogeneity. As requested also by reviewer 1, the means and SD for reporter-induced intracellular Salmonella were calculated for three replicates and added as additional panels for Fig. 1, 6, and 9. See also response to reviewer 2.

2. The authors have shown that gentamicin protection assay indicates no contribution of T3SS-2 to replication of S. Typhi in RAW264.7 and U937 cells (Fig. 7). From the graph it’s hard to know either S. Typhi wild-type can’t replicate in the macrophages unlike S. Typhimurium (or rather replicate like S. Typhimurium ssaV) or S. Typhi can replicate intracellularly regardless of the presence of T3SS-2. To clear it, the authors should repeat the experiment Fig 8 and show growth rate (1h/24h) of S. Typhimurium and S. Typhi in each tissue culture beside the flow data plots.

Response: We opted for analyses of intracellular proliferation by conventional gentamicin protection assays (Fig. 7) analysing the ensemble, and the flow cytometry approach inf Fig. 8 for analyses of single host cells. As the approaches are rather distinct in method and interpretation, we consider it more appropriate to keep the data sets and legend separate. The data shown in Fig 8 and corresponding S7 Fig should provide the requested information

3. The ssaG expression of S. Typhi in RAW264.7 looks better than that of S. Typhimurium (Fig. 1D), but no effect on S. Typhi intracellular replication (Fig. 7). Some explanation should be required for the gap.

Response: Thank you for the comment. We could explain this by the difference between induction of expression and the low levels of effector translocation. We picked up this point in the discussion, section ‘Low intracellular activity of TS’, lines 558 ff.-

4. Translocation of sifA (sifA::M45) by the typhoidal Salmonella serovars is required (Fig. 3) because SIF formation is focused in this study. It remains unclear the reduction of SIF formation observed by infection with S. Typhi and S. Paratyphi A (Fig. 4) is resulted in reduction of sifA expression and translocation.

Response: We analysed translocation of SifA-M45 by immunolabeling. However, due to the rather low signal intensities for SifA, the data remained inconclusive. Additional analyses of other effector proteins indicated severely reduced amounts of translocated effector proteins (Fig. 3D). As an alternative means of analysis of SifA, were generated a dual-fluorescence reporter with the promoter of sifA and analysed induction of STM, STY and SPA. The new data set Fig. 4C shows delayed expression of sifA and lower levels of expression of sifA in STY and SPA compared to STM. We consider this new data as supportive for the model of delayed and reduced expression of SPI2-T3SS functions in TS.

5. The images in Fig 4A show SIF at 8h post infection, but graphs in Fig. 4B show SIF at 16h and 24h post infection. Authors should the frequency of SIF positive cells in the graph at 8h instead of one of later time points.

Response: We removed the quantification of SIF for 16 h and 24 h time points and analysed SIF frequency at 8 h p.i. in order to match the micrographs displayed (revised Fig. 4.

6. Some sections don’t have authors’ conclusion from consequence of each result. That may make readers get confused. For instance, in the section “SCV integrity and cytosolic access of intracellular STY and SPA”, author should give any opinion why S. Typhi ∆sifA strain didn’t reduce its SCV integrity unlike S. Typhimurium ∆sifA strain, and how it is relating with other obtained results.

Response: We added a concluding paragraph for this section. Please note that concluding statements are already given for the key observations reported.

No point 7

8. Biosynthetic capability of intracellular Salmonella is required for T3SS-2 activity (Fig. 9)? The author concluded the typhoid Salmonella serovars have lower capacity than S. Typhimurium to maintain a biosynthetic active sated over prolonged presence in host cells (lane 327). But it remains unclear how T3SS-2 is involved in the activity. The author should determine if a S. Typhimurium T3SS-2 mutant abolished the activity.

Response: We performed the suggested experiment by infection of various host cell types with STM WT and SPI2-T3SS deficient mutant, and determined sfGFP expression upon induction by AHT addition. The new data set is added as S8 Fig, and the observations are referred to in line 527 ff. We did not observed effects of T3SS-2 deficiency on biosynthetic activity of STM in phagocytic cell. The biosynthetic activity of the STM ssaV strain was only reduced at late stages of infection in HeLa cells.

Part III – Minor Issues: Editorial and Data Presentation Modifications
Please use this section for editorial suggestions as well as relatively minor modifications of existing data that would enhance clarity.

Reviewer #1: •

Throughout the manuscript, the authors should do a better job explaining the rationale for using specific effector mutants (e.g. delta-ssaV, delta-ssaR, delta-ssaK, etc.).

Response: All of the mutations result in loss of function of the SPI2-T3SS. As STY and SPA strains resulted from in Infect-ERA consortium, different deletions were used in the various serotypes. We added a statement on this observation, line 206.

• Lines 162-163: “The amounts of effector proteins (…) than for STM-infected host cells (data not shown).” Please, do show these data. Similar for the following sentence “Also, the frequency (…) was lower.” This statement seems not to be supported by any of the presented data.

Response: We addressed this point by microscopy-based quantification of translocated effector proteins on the level of single host cells infected by STM, SPA or STY. The new data set is added as Fig. 3D. The data demonstrate the generally lower levels of effectors sseF, sseL, sseJ, and pipB2 in cells infected by STY or SPA. The removed the statement of different frequencies of cells positive for effector translocation.

• Lines 168-169: “Translocation of SseJ is possible in STY and SPA, although sseJ is a pseudogene in these serovars (…)” Please elaborate, e.g. which part of the gene is mutated in typhoidal compared with nontyphoidal Salmonella? Would this mutation be expected to prevent transcription/translation or result in the production of a shorter protein? Can the authors provide additional (more convincing) images that this effector is indeed produced and translocated by SPA and STY (there is barely any signal visible in current Fig. 3 for this effector).

Response: Please note that episomal encoded sseJ of STM was used to test translocation of epitope-tagged SseJ by STY and SPA. Thus, we cannot make statements on synthesis and translation of truncated form of SseJ by STY and SPA. The levels of translocated effector proteins in STY or SPA infected cells are low, please refer to the new quantitative data Fig. 3D. The micrographs were generated with comparable settings. Artificial enhancement of signal intensities for effector protein straining would suggest higher levels of translocated and thus was avoided. Please also refer to the high-resolution images.

• Lines 192-193: “Since imaging (…) not assessed.” Please explain this in more detail as it may not be obvious for all readers why fixation would prevent SIF detection in macrophages.

Response: Reference to earlier work describing fragmentation of SIF upon fixation is given, line 292.

• Lines 216, 218: The acronyms “CLEM” and “TEM” need to be introduced.
• Line 268: “(…) information and functionally of (…)” Please correct.
• Line 277: “(…) and the deficient in proliferation (…)” Please correct.
• Line 281: The acronym “FP” needs to be explained.

Response: Text corrected for all points mentioned

• Line 294-296: “We anticipated that (…)” I am not sure if I understand the authors’ hypothesis. Hasn’t it been shown that in vivo Salmonella persisters retain metabolic activity (see work from Helaine lab)?

Response: Additional explanation and reference to related studies has been added, line 474 ff.

• Line 419: “(…) a recently organoid model (…)” Please correct.

Response: Corrected

• Fig. 1A: The main conclusion the authors draw from these data is that all the tested Salmonella serovars activate SPI-2 in PCN-P (see line 116 in the Results section). What is not mentioned, however, is that the magnitude of SPI-2 induction (intensity of the reporter signal) per bacterial cell is substantially lower for SPA and STY as compared with STM. Thus, it seems that not only the proportion of SPI-2+ bacteria is lower in the typhoidal serovars (panels B-D), but also the expression levels of SPI-2 genes within the SPI-2+ subpopulation. Can the authors please comment?

Response: Thank you, well spotted. We amended the results section for Fig. 1 to describe this observation in more detail, line 132 ff.

• Fig. 1D: As judged by eye, the shoulder of the red graph (STY WT) that extends into the “induced” population in the presence of IFNg implies a smaller value than the reported 51.45%. Can the authors comment, please?

Response: We check the data of the triplicate experiments and corrected the percentages. As indicated by the means and SD for the triplicates, the variation between induced Salmonella in activated RAW264.7 macrophages was rather high.

• Fig. 3: The effects seem relatively subtle. Can effector translocation please be quantified over multiple cells (e.g. how many of the Salmonella-infected cells were stained positive for the respective effectors?) and these data be plotted here, rather than/in addition to the single (representative) microscopy image? In addition, inclusion of STM in these experiments would be appreciated, as it would allow for direct comparison of the results between all three serovars. (E.g. in regard of my above comment: How would translocation compare between typhoidal (pseudogenized) and nontyphoidal SseJ?)

Response: We addressed this point by adding quantitative data for signal intensities of various effectors translocated by STM, STY and SPA. This is new Fig. 3D. While individual host cells indicate that effector translocation in general low in STY and SPA infected cells, quantification shows that overall levels are rather low.

SseJ translocation was quantified, as for other effectors, by introducing epitope-tagged SseJ of STM encoded by a plasmid. Thus, translocation is independent from the pseudogene sseJ in TS.

• Fig. 4A: Please explain in the figure legend what the insets and yellow arrowheads refer to.

Response: The legend has been revised accordingly to explain these elements.

• Fig. 4B: The figure legend informs about the statistical analysis (n.s.; *, **). However, not all bars in this panel have any statistical label. Can the authors please add this?

Response: The statistical analyses through the manuscript were done for comparison of STY to STM and SPA to SPA. We consider this comparison as most relevant.

• Fig. 6: As the authors rightfully mention (lines 249-250) STM delta-sifA mutants are deficient in maintaining SCV integrity, resulting in their enhanced cytosolic exposure. As inferred from panel B, it seems as if SifA had the opposite effect in STY inside HeLa cells (where sifA deletion apparently DEcreased cytosolic exposure). Can the authors please comment?

Response: Please consider that the sifA mutant phenotype is apparent in host cell that permit Salmonella proliferation. In absence of detectable intracellular proliferation, the need for vesicle recruit to allow SCV extension may be far less pronounced.

• Fig. 7: Rather than plotting replication relative to the WT, I’d prefer if replication values were plotted relative to the respective 1 h values. This way, differences between the different cell lines and serovars could be deduced.

Response: The presentation of intracellular proliferation as percentage of WT was chosen because means and standard deviations of triplicate experiments are shown. Given the variation between rates of invasion or phagocytosis between experiments, especially to TS, calculation of means from absolute values would generate unnecessary high variations.

• Fig. 8A is confusing: It looks as if the delta-ssaV mutant (at 1 h p.i.) had an increased SSC-H signal compared to the WT (i.e., the yellow/orange scatters extend above the red scatters). Additionally, at 24 h the ssaV mutant (green dots) seems to extend to the right (mCherry axis) as compared to the WT (blue cloud) despite the reported numbers suggesting that the mCherry signal of the ssaV mutant is only half the signal of the WT at 24 h. Can the authors comment? I wonder if this representation really is the best way of visualizing the results. Also, Salmonella reporter plasmids have been reported that allow for a more accurate quantification of (intracellular) replication (PMIDs: 20133586; 25126781). The authors may want to switch to one of these systems to measure intracellular proliferation more precisely.

Response: Please also see S7A Fig for the representation of single scatter plats. The key message is the increase of mCherry intensity over time in a SPI2-T3SS dependent manner. Over the interval of 24 h, a certain level of intracellular proliferation of ssaV mutant strains can be observed, especially by cytosolic hyperreplication. This may explain the small population with very high mCherry intensities. Currently, we cannot explain the differences in SSC values. For these experiments, we used a single FP and mCherry as very fast maturing FP. This was done to provide maximal correlation between fluorescence signals and bacterial burden of host cells. The fluorescence dilution approach used in the mentioned studies may as well be applied. We used the fluorescence dilution approach in other studies for STM and will consider this for future work on TS.

• Fig. 9A: In case of SPA, there seems to be heterogeneity in AHT responsiveness in vitro (which is absent from STM and STY). Is this worth mentioning?

Response: Yes, indeed. This is in line to heterogeneity observed in Fig. 1. We added a section on this observation, line 482.

• Fig. 9C, D: For both SPA and STY there seems to be some kind of oscillation in AHT responsiveness. That is, inside both HeLa and U937 cells the induced population was high at 8 h p.i., then dropped at 16 h p.i., and increased again at 24 h p.i. Do the authors have any explanation for this phenomenon?

Response: Sorry, no explanation sufficient for inclusion in the manuscript. One might consider changes in host cell viability and antimicrobial activities as factors acting on biosynthetic capacity of intracellular Salmonella. STY and SPA entering persister status may switch back to vegetative growth at later time points of infection. This is probably worth in-depth analyses by subsequent work.

Reviewer #2:

1. L27. Not all S. Typhimurium are associated with a broad host range and so consider '....many S. enterica serovar Typhimurium (STM)....'.

Response: Text adjusted

2. L35. Suggest being precise about which single cell analyses were used in this study, 'such as' suggest these are only examples and makes the reader believe there may be others.

Response: Text adjusted

3. L51. Commonly, poor infrastructure for sanitation rather than simply hygiene are implicated in TS infections.

Response: Text adjusted

4. L79. Typo 'SP2-T3SS'

Response: Text adjusted

5. 82-84. Suggest discussing the humanised mice model described by Karlinsey et al, 2019 here too.

Response: Reference to this work is given now.

6. L121. Please indicate the type of cells HeLa and U937 are as is done for RAW264.7 cells. Most will know that HeLa are epithelial but should be added anyway. U937 are less familiar.

Response: Text adjusted

7. L171. Please specify if these observations are in vivo or in cultures cells and if these were phagocytic or non phagocytic cells , or reported in all?

Response: Text adjusted to provide details on the infection models used.

8. L175. Typo, should be 'completely'?
9. L294. suggest '...capable of protein....’
10. L390. Typo, should be 'subtle'?
Response: Text adjusted

11. L531-553. Please describe the statistical analysis of the cytometry data.

Response: The statistical methods have been added. For FC analyses in Fig. 1, 6, and 9, and new the percentage of induced bacteria. Throughout the study, One-way ANOVA as used.

Reviewer #3:

1. Author should describe the origin of each tissue culture (lane 122) and mention why those tissue cultures are chosen for the study.

Response: Further justification for use of the cells lines is given, line 163 ff.

2. Author should explain a reason why only RAW264.7, but not other macrophage cell lines, needed to get primed by IFN-gamma (lane 132). I am also confused whether or not authors used the primed RAW264.7 cells for latter experiments.

Response: RAW26.7 are routinely used without IFN-gamma activation as resting phagocytes with lower antimicrobial capacity, and with IFN-gamma activation to increase antimicrobial functions. If not indicated, macrophages were using without IFN-gamma activation. For clarity, this is added to the legends.

3. In human macrophages, all serovars didn’t show expression of ssrB unlike in U937. The authors should give any suggestions for this point why T3SS-2 expression was different in between human macrophage and U937.

Response: In case the difference in expression of PssaG::sfGFP by WT are meant by the reviewer: We conclude that primary macrophages have high antimicrobial capacity compared to cell line macrophages. The primary human macrophages used here were predominantly M1 polarized. The added a section with this explanation, lines 181-183

4. Lane 192 “Since imaging was…” means SIF is not observed in fixed macrophages infected with Salmonella? Any reference?

Response: A more detailed explanation is now given, this point was also raised by reviewer 1. We not address SIF formation in macrophages by STY and SPA in this study, focus was rather to test if STY and SPA are capable in SIF induction per se. (line 292).

5. S. Typhimurium wild type showed reduction of SCV integrity in HeLa but not in U937 over the course of infection (Fig. 6). How about in RAW264.7 cells? I wonder if S. Typhimurium doesn’t reduce the SCV integrity specifically observed in macrophages.

Response: We reported on STM cytosolic access in RAW264.7 in an early publication (PMID: 31872936). The conclusion was that cytosolic presence of Salmonella in macrophages in only transient and either induces pyroptosis of host cells, or xenophagic clearance of Salmonella. What is remarkable is the high percentage of U937 cells with cytosolic STM sifA. This may be due to an altered pyroptotic response of U937, but has not been further investigated.

6. Some abbreviations are unknown for (“FC” on lane 259; “FP” on lane 281). Bacterial names in the legends of Figs S4-S6 are not consistent with the bacterial names of images. Lane 711, “in revision”? They are needed to be fixed.

Response: Thank you for pointing out these errors. The abbreviations are now introduced at first occurrence, and the suppl. legends and legends corrected. The ‘in revision’ manuscript is now published and fully cited.
